# Management and outcome of traumatic subdural hematoma in 47 infants and children from a single center

**DOI:** 10.1007/s00508-020-01648-3

**Published:** 2020-04-24

**Authors:** Harald Binder, Thomas M. Tiefenboeck, Marek Majdan, Micha Komjati, Rupert Schuster, Stefan Hajdu, Johannes Leitgeb

**Affiliations:** 1 Department of Orthopaedics and Trauma Surgery, Division of Trauma Surgery, Medical University of Vienna, Waehringer Guertel 18–20, 1090 Vienna, Austria; 2 Department of Orthopaedics, Sacred Heart Hospital of Jesus, Vienna, Austria; 3 International Neurotrauma Research Organization, Vienna, Austria

**Keywords:** Traumatic brain injury, Prognostic factors, Outcome, Glasgow coma scale, Rotterdam score

## Abstract

**Background:**

Traumatic brain injury (TBI) is a frequent cause of mortality and acquired neurological impairment in children. It is hypothesized, that with the adequate treatment of SDH in children and adolescence, excellent clinical and functional outcomes can be achieved. The aim of this study was to present the severity and outcome of traumatic SDH in children and adolescence as well as to analyze differences between patients treated surgically and conservatively.

**Methods:**

In this study 47 infants and children with a subdural hematoma (SDH) were treated between 1992 and 2010 at a single level-one trauma center. Data regarding accident, treatment and outcomes were collected retrospectively. To classify the outcomes the Glasgow outcome scale (GOS) scores at hospital discharge and at follow-up visits were used. Severity of SDH was classified according to the Rotterdam score.

**Results:**

In total, 47 cases were treated (21 surgically, 26 conservatively), with 10 patients needing delayed surgery. Overall, 89% of the patients were able to leave hospital, 5 patients died, 2 patients (5%) within 24 h, another 2 (5%) after 48 h and 1 (2%) within 7 days. In 25 patients (53%) a good recovery was recorded at the last follow-up visit. Outcome was mainly influenced by the following factors: age, severity of TBI, and neurological status. Overall, in 70% good clinical and neurological outcomes could be achieved.

**Conclusion:**

The results of this study confirmed that pediatric SDH is a rare, but serious condition. Despite a poor prognosis, most patients could be treated with good outcomes, given that the choice of treatment is correct.

**Trial registration:**

Research registry 2686

**Electronic supplementary material:**

The online version of this article (10.1007/s00508-020-01648-3) contains supplementary material, which is available to authorized users.

## Introduction

Trauma is still the leading cause of death in children. Severely injured young patients often present a traumatic brain injury (TBI) which is a frequent cause of mortality [1, 2]. Despite this, pediatric TBI is a very rare injury and physicians often rely on their clinical experience as opposed to clinical literature when making treatment decisions [3]. Acute subdural hematoma (SDH) in children, especially in infants and toddlers, is relatively uncommon, with reported incidences of 20–25 cases per 100,000 children [4]. Between 42 and 82% of infantile SDH are believed to result from child abuse (CA), which is considered to bear a poorer prognosis [5, 6]. Due to its rare occurrence [7, 8] pediatric SDH is a challenging condition to treat. The outcome of pediatric subdural hematoma is worse than that of epidural hematoma, with mortality rates ranging from 42–90%; subdural hematoma associated with child abuse is associated with a 20% mortality rate and a 50% rate of neurological morbidity [9].

Since the implementation of computed tomography (CT), accurate detection of SDH has become easier. However, variations in treatment and outcome make it difficult to draw a clear picture of traumatic SDH in children and infants [10, 11].

The aim of this study was to compare operative versus conservative treatment in infants and children with traumatic SDH with regard to severity and outcome.

## Methods

### Data collection

In a retrospective single center study, all patients with traumatic SDH under the age of 16 years treated between 1992 and 2010 were included. In total, 47 patients were included. The following patient data were extracted and presented in charts: patients demographics (age, sex), cause of injury, injury severity (Injury Severity Score (ISS), Glasgow Coma Scale (GCS), and additional injuries), CT findings, treatment modalities (surgical vs. conservative, types of surgery, additional treatment) and outcomes (GOS) at discharge and at follow-up.

### Treatment procedures

Treatment procedure in all patients included rapid examination by an emergency physician (pediatric GCS and pupillary reactivity). Further medical treatment included rapid sequence intubation, ventilation, treatment of hemorrhage, treatment of associated substantial extracranial injury and fluid resuscitation, as deemed appropriate.

In the next step a CT scan was performed in every patient. Examination was performed by a trauma team of anesthesiologists, trauma surgeons and/or neurosurgeons, radiologists and nurses. Depending on the CT result, further treatment, including surgery and/or ICU admission, was undertaken. Trauma surgeons carried out neurosurgery (burr hole trepanation, craniectomy and craniotomy) in collaboration with neurosurgeons for complex issues.

Prehospital parameters and treatment were documented by paramedics. The CT findings were analyzed by a team of trauma surgeons, neurosurgeons and radiologists specialized in trauma diagnostics. In all patients with hemorrhage the Rotterdam score was calculated [12].

### Data analysis

Data analysis focused on surgically versus conservatively treated patients with SDH. Patients were divided into an operated group (at least one cranial surgery) and conservative group. The following parameters were analyzed between the two groups: demographic characteristics, injury cause, severity and outcome.

In cases of continuous variables medians with respective interquartile ranges were calculated and used as central measures. In cases of categorical variables total values with corresponding percentages were calculated as measures of frequency. In order to estimate the population proportions, 95% confidence intervals were calculated wherever percentages were used.

Statistical analyses were performed using the R project statistical environment (R package version 0.4, Bell Laboratories [formerly AT&T, now Lucent Technologies]; Hewson, B. 2015). A *p*-value of <0.05 was considered statistically significant.

## Results

An overview of patient demographics and injury causes is presented in Table 1.

**Table 1:** Demographic characteristics of patients and characteristics of trauma in conservatively and surgically managed pediatric TBI patients with SDH

Measure	Conservative	Delayed surgery	Surgical	Total	*P* value
*Total N (%)*	*26 (55%)*	*10 (21%)*	*11 (23%)*	*47*	**–**
*Age (years median, IQR)*	3.5 (1–4.9)	4 (1–7.25)	2 (1.5–8.5)	4 (1–8.5)	0.911
*Sex (N, % male)*	12 (46%)	7 (70%)	4 (36%)	23 (49%)	0.279
*Trauma mechanism (N, %)*					
Battered child syndrome	2 (8%)	2 (20%)	0	4 (9%)	0.454
Fall (50–150 cm)	7 (27%)	3 (30%)	0	10 (21%)	–
Fall (<50 cm)	1 (4%)	0	0	1 (2%)	–
Fall (>150 cm)	7 (27%)	2 (20%)	7 (64%)	16 (34%)	–
Traffic accident	4 (15%)	1 (10%)	1 (9%)	9 (19%)	–
Sports	4 (15%)	2 (20%)	3 (27%)	6 (13%)	–
Other	1 (4%)	0	0	1 (2%)	–
*Polytrauma (N, % Yes)*	14 (54%)	10 (100%)	11 (100%)	35 (75%)	<0.01

*IQR* interquartile range

The median age was 4 years in the operated group and 3.5 years in the conservative group. Regarding gender, male was shown to be prevalent in the conservative group. In the operated group falls from over 150 cm and traffic accidents were more frequent compared to falls between 50–150 cm, which were more common in the conservative group. Female cases of SDH were more frequently observed in toddlers compared to babies, while in the male group no differences were observed. It was an increasing number of high impact traumas with increasing age detected, which suggests an association. (Table 2).

**Table 2:** Injury severity indicators in conservatively and surgically managed pediatric TBI patients with SDH

Measure/Treatment type	Conservative	Delayed surgery	Surgical	Total	*P* value
*Total N (%)*	*26 (55%)*	*10 (21%)*	*11 (23%)*	*47*	*–*
*Symptoms indicating TBI (N, % Yes)*					
Swellings	13 (50%)	4 (40%)	4 (36%)	21 (45%)	0.706
Nausea	6 (23%)	4 (40%)	0	10 (21%)	<0.01
Vomiting	6 (23%)	3 (30%)	3 (27%)	12 (26%)	0.303
*Unconsciousness (N, % present)*	11 (42%)	5 (50%)	10 (91%)	26 (55%)	<0.01
*Neurological status (N, %)*					
Normal	12 (46%)	1 (10%)	0	13 (28%)	<0.01
Somnolent	9 (35%)	6 (60%)	3 (27%)	18 (38%)	–
Comatose	5 (19%)	3 (30%)	8 (73%)	16 (34%)	–
*Pupils (N, %)*					
Both reactive	21 (81%)	5 (50%)	5 (46%)	31 (66%)	0.11
One reactive	3 (12%)	4 (40%)	3 (27%)	10 (21%)	–
None reactive	2 (8%)	1 (10%)	3 (27%)	6 (13%)	–
*ISS (median, IQR)*	12.5 (9–16)	25 (25–25)	25 (20–31.5)	16 (9–25)	<0.001
*First pediatric GCS (median, IQR)*	3.5 (1–8.75)	4 (1–7.25)	2 (1.5–8.5)	4 (1–8.5)	0.911
*Admission pediatric GCS (median, IQR)*	3.5 (1–8.75)	4 (1–7.25)	2 (1.5–8.5)	4 (1–8.5)	0.912
*Vertebral fracture (N, %)*					
Neck Region	1 (4%)	0	0	1 (2%)	0.669
Thorax Region	0	0	0	0	–
*Additional Injuries (N, %)*					
Upper extremity Fracture	1 (4%)	1 (10%)	1 (9%)	3 (6%)	0.256
Lower Extremity Fracture	1 (4%)	1 (10%)	3 (27%)	5 (10%)	0.134
Injury to thoracic region	1 (4%)	1 (10%)	4 (36%)	6 (12%)	0.182
Injury to abdominal region	0	0	2 (18%)	2 (4%)	0.032
*Rotterdam CT Score (N, %)*					
1	2 (8%)	0	0	2 (4%)	0.049
2	12 (46%)	2 (20%)	1 (9%)	15 (32%)	–
3	8 (31%)	3 (30%)	4 (36%)	15 (32%)	–
4	3 (12%)	4 (40%)	3 (27%)	10 (21%)	–
5	1 (4%)	0	3 (27%)	4 (9%)	–
6	0	1 (10%)	0	1 (2%)	–

*TBI* traumatic brain injury, *SDH* subdural hematoma, *ISS* injury severity score, *IQR* interquartil ratio, *GCS* Glasgow coma scale, *CT* computed tomography

**Fig. 1:**
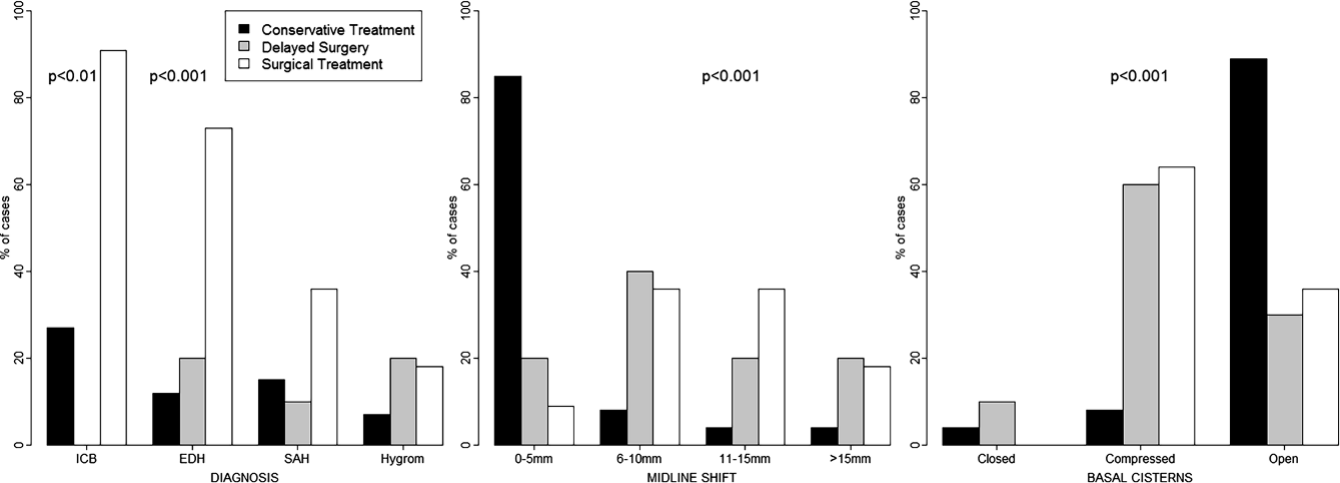
Conservative versus surgically treated patients with respect to diagnosis, midline shift and compression of the basal cisterns. *ICP* intracranial pressure, *EDH* epidural hematoma, *SAH* subarachnoidal hematoma

All operated patients had additional severe injuries of which 71% were unconscious, 52% were comatose and the same proportion had at least 1 unreactive pupil. The median ISS was significantly higher in the operated group (median 25 vs. 12.5). A Rotterdam CT score of 3 or higher was observed in 85% of patients in the operated group compared to 47% in the conservative group. The CT findings (presence of additional diagnoses, midline shift and/or compression of basal cisterns) comparing surgical and conservative treatment are shown in Fig. 1., suggesting more severe injuries in the operation group. An overview of treatment factors is shown in Table 3.

**Table 3:** Treatment factors in conservatively and surgically managed pediatric TBI patients with SDH

Measure/treatment type	Conservative	Delayed surgery	Surgical	Total	*P* value
*Total N (%)*	*26 (55%)*	*10 (21%)*	*11 (23%)*	*47*	–
*Transport (N, % Air)*	7 (27%)	1 (10%)	6 (55%)	14 (30%)	0.018
*Intubation (N, %)*	5 (19%)	4 (40%)	7 (64%)	16 (34%)	0.031
*X-ray done (N, %)*	21 (81%)	5 (50%)	8 (73%)	34 (72%)	0.181
*CT scan done (N, %)*	26 (100%)	10 (100%)	11 (100%)	47 (100%)	–
*MRI done (N, %)*	6 (23%)	1 (10%)	1 (9%)	8 (17%)	0.469
*ICU days (N, %)*					
No admission	16 (62%)	0	0	16 (34%)	0.016
≤10 days	9 (35%)	7 (70%)	5 (45%)	21 (45%)	
11–20 days	0	1 (10%)	1 (9%)	2 (4%)	
21–30 days	1 (4%)	1 (10%)	3 (27%)	4 (9%)	
Over 30 days	0	1 (10%)	3 (27%)	4 (9%)	

*TBI* traumatic brain injury, *SDH* subdural hematoma, *CT* computed tomography, *MRI* magnetic resonance imaging, *ICU* Intensive care unit

**Fig. 2:**
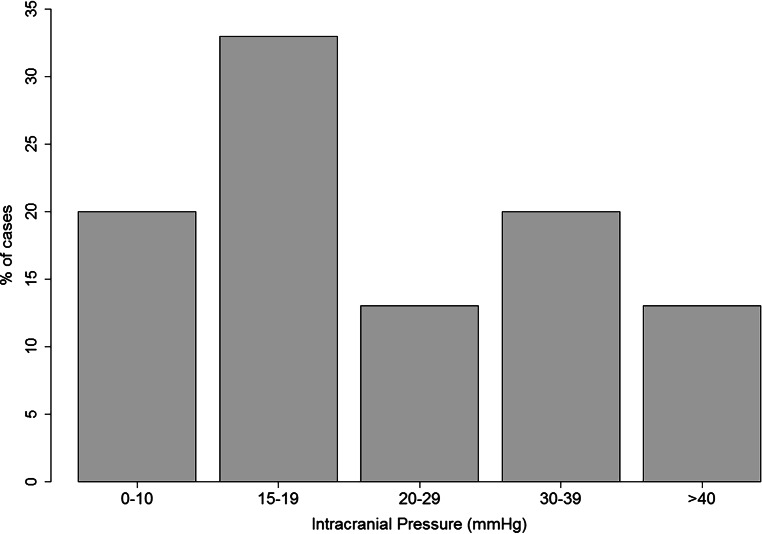
Intracranial pressure (in mm Hg) recorded at time of surgery

There was no statistically significant difference regarding the mode of transport (air vs. ground) in the two groups; however, patients in the operative group were significantly more often intubated in the field (52% vs. 19%) and had a significantly longer stays in the ICU (23% stayed longer than 20 days vs. 4% in the conservative group). In the operated group 50% of patients were treated within 1h after admission, whereby 25% needed a second surgical intervention and 2 patients (10%) even required 4 operations. In this patient collective two factors accounting for delayed surgery were found: (1) severely injured patients and (2) failed conservatively managed patients (for details see Table 2 and 4). In total ICP was recorded in 71% of patients (Table 4, Fig. 2).

**Table 4:** Treatment factors in surgically managed pediatric TBI patients with SDH

Measure	Delayed surgery	Surgical	Total	*P* value
*Surgery time (N, %)*	*10 (48%)*	*11 (52%)*	*21*	*–*
<1 h	0	11 (100%)	11 (52%)	<0.001
<24h	7 (70%)	0	7 (33%)	–
<1 week	1 (10%)	0	1 (5%)	–
Delayed after 4 h	2 (20%)	0	2 (10%)	–
*Number of TBI surgeries within 24* *h (N, %)*				
One	9 (90%)	10 (91%)	19 (90%)	1
Two	0	2 (19%)	2 (10%)	–
*Overall number of TBI surgeries (N, %)*				
One	8 (80%)	6 (55%)	14 (66%)	0.023
Two	0	5 (46%)	5 (24%)	–
Four	2 (20%)	0	2 (10%)	–
*Other surgery (N, %)*				
Multiple surgery	0	1 (9%)	1 (5%)	1
Osteosynthesis (external)	1 (10%)	1 (9%)	2 (10%)	–
Mediastinal drain	0	1 (9%)	1 (5%)	–
*Parenchymal ICP monitor (N, %)*	5 (50%)	10 (91%)	15 (71%)	0.063
*Ventricular drain (N, %)*	1 (10%)	0	1 (5%)	0.472
*Days of ICP monitoring (N, %)*				
≤10 days	4 (40%)	6 (55%)	10 (47%)	0.145
11–20 days	1 (10%)	1 (9%)	2 (10%)	–
21–30 days	0	2 (18%)	2 (10%)	–
30+ days	0	1 (9%)	1 (5%)	–
None	5 (50%)	1 (9%)	6 (29%)	–

*TBI* traumatic brain injury, *SDH* subdural hematoma, *ICP* Intracranial pressure

Removal of ICP monitoring is standardized when ICP parameters show no value >10 mm Hg in 24 h. This is part of the policy of the investigating center, which aims to minimize the risk of postoperative infections.

Regarding outcome a significant difference in the evaluated GOS at hospital discharge between the two groups was found; however, at the latest follow-up no differences in GOS were found (Table 5).

**Table 5:** Outcomes in conservatively and surgically managed pediatric TBI patients with SDH

Measure/treatment type	Conservative	Delayed surgery	Surgical	Total	*P* value
Total *N* (%)	26 (55%)	10 (21%)	11 (23%)	47	
*GOS hospital discharge (N, %)*					0.016
Death	1 (4%)	1 (10%)	3 (27%)	5 (11%)	
Vegetative state	0	0	1 (9%)	1 (2%)	
Severe disability	0	1 (10%)	0	1 (2%)	
Moderate disability	4 (15%)	4 (40%)	5 (45%)	13 (28%)	
Good recovery	20 (77%)	3 (30%)	2 (18%)	25 (53%)	
Unknown	1 (4%)	1 (10%)	0	2 (4%)	
*GOS follow-up (N, %)*					0.084
Death	1 (4%)	1 (10%)	3 (27%)	5 (11%)	
Vegetative state	0	0	1 (9%)	1 (2%)	
Severe disability	0	0	0	0	
Moderate disability	1 (4%)	2 (10%)	0	3 (6%)	
Good recovery	20 (77%)	5 (50%)	7 (64%)	32 (68%)	
Unknown	4 (15%)	2 (20%)	0	6 (13%)	
*Time of death (N, %)*					0.067
Survivors	25 (96%)	9 (90%)	8 (73%)	42 (89%)	
Within 24 h	1 (4%)	0	1 (9%)	2 (4%)	
Within 48 h	0	0	2 (18%)	2 (4%)	
Within 7 days	0	1 (10%)	0	1 (2%)	
*Cause of death (N, %)*					0.088
Survivors	25 (96%)	9 (90%)	8 (73%)	42 (89%)	
Brain death	1 (4%)	0	2 (18%)	3 (6%)	
Cardiovascular failure	0	1 (10%)	0	1 (2%)	
Pulmonary failure	0	0	1 (9%)	1 (2%)	

*GOS* Glasgow Outcome Scale, *TBI* Traumatic brain injury

Most patients showed a favorable outcome (moderate disability or good recovery) both at discharge (53%) and at follow-up (68%). The proportion of unfavorable outcome (death, vegetative state and severe disability) was higher in the operative group, especially at hospital discharge. In the surgically treated group, 4 patients (19%) died, 2 within 48 h and 2 within 7 days after hospital admission. In the conservatively treated group, 1 patient (4%) died within 24 h after hospital admission. The overall mortality rate of SDH in infants and children in the sample was 11%. In 52% of the patients educational achievements could be evaluated at the latest follow-up visit. In 61% of the patients a normal academic performance could be achieved. With respect to the neurocognitive function, patients who underwent surgery tended to have an unfavorable outcome.

## Discussion

Summarizing the results, the following factors were associated with surgical treatment: low GCS scores, high ISS, worse CT findings, radiologically detectable cerebral injuries, a midline shift of more than 5 mm, compressed or closed basal cisterns and neurological deficits; however, about 70% of the patients achieved a good recovery at final follow-up. In 61% of the patients a normal academic performance could even be reached.

The controversies about the treatment mode (surgery or conservative treatment) in pediatric SDH have not been resolved. There are still problems with hematoma removal in infants due to the technical difficulty of full evacuation and the potential danger of iatrogenic hemorrhage [13]. One aspect could help such decisions: while secondary risk factors influence the outcome significantly in adult brain-trauma patients [14], this effect is less apparent in children [15]. This is also supported by the data of the performed study.

Bullock et al. [16] and Rivas et al. [17] described worse outcomes in surgically treated cases with EDH associated with SDH, which also could be proven. In the recent study, 10 patients (48%) with EDH were operatively treated and 3 patients (12%) were conservatively treated. 

Conservative management was mostly performed in patients without neurological symptoms, midline shift less than 5 mm in the CT and open basal cisterns, presenting with good neurological outcome.

This is in accordance with the findings in other similar cohorts [15, 16].

The hospital mortality of SDH is described as being 11–36% in the literature [4], which corresponds well to the rate of 11% in the recent study.

The cause of death is additionally influenced by cardiovascular and pulmonary failure, which were the reasons of death in two operated children with polytrauma.

Although SDH is often regarded as a manifestation of child abuse [8, 9, 18–20], only 4 cases (9%) were detected. All of them were under the age of 4 years, with the two cases who died of acute brain death being under the age of 1 year serving as good examples for the poorer prognosis in infantile SDH resulting from child abuse [18]. Although only 9% suffered from SDH due to child abuse, the absence of a history of significant accidental trauma necessitates a full investigation of maltreatment, which is also recommended in the literature [4]. Summarizing the negative prognostic factors for survival, the following were found: surgically treated SDH combined with EDH, polytrauma, diffuse swelling as a potent precursor of cerebral herniation and SDH resulting from child abuse. No statistically significant difference concerning negative clinical results in relation to multiple surgical procedures could be detected.

There are considerable limitations to this study: it had a retrospective design and a relatively small cohort of patients, which is a consequence of the rather rare occurrence of these specific types of trauma, but nevertheless it limits the generalizability of the findings. The study represents a homogeneous patient group with the main outcome parameters reported and therefore is able to make a valid conclusion.

## Conclusion

This study confirmed that pediatric SDH is a rare, but serious condition. Despite a poor prognosis, most patients could be treated with good outcomes, given that the choice of treatment was correct. In practice, the treatment decisions are to a large extent left to the physician. This could be overcome by clear treatment guidelines, the definition of which requires more studies similar to this one, ideally with data sets and a longer follow-up period.

## Caption Electronic Supplementary Material

STROBE Guidelines

## Abbreviations

AIS: Abbreviated injury scale
CA: Child abuse
CT: Computed tomography
EDH: Epidural hematoma
GCS: Glasgow coma scale
ICP: Intracranial pressure
ICU: Intensive care unit
ISS: Injury severity score
MRI: Magnetic resonance imaging
SDH: Subdural Hematoma
TBI: Traumatic brain injury
