# Supplementary material for: Management and outcome of traumatic subdural hematoma in 47 infants and children from a single center
Source: Wien Klin Wochenschr. 2020 Apr 24;132(17):499–505. doi: 10.1007/s00508-020-01648-3 (PMC7518988; doi:10.1007/s00508-020-01648-3)
Supplement: Supplementary file 1 — STROBE Guidelines [file 508_2020_1648_MOESM1_ESM.docx]

STROBE Guidelines

To be used by authors of all observational clinical studies. For this purpose a cohort study (the term used by STROBE) is considered a longitudinal study typically reporting outcomes of treatment in one or more cohorts; a case-control study is one identifying factors in outcomes; a cross-sectional study is one to identify the prevalence of factors or characteristics in a population at a single point in time.

This checklist table is modified from The STROBE Initiative, [www.strobe-statement.org](http://www.strobe-statement.org) and should be filled and submitted within the electronic submission

|  | Item No | Recommendation | Please insert check where included or N/A where not applicable |
| --- | --- | --- | --- |
| **Title and abstract** | 1 | (*a*) Indicate the study’s design with a commonly used term in the title or the abstract | check |
|  |  | (*b*) Provide in the abstract an informative and balanced summary of what was done and what was found | check |
| Introduction | | |  |
| Background/rationale | 2 | Explain the scientific background and rationale for the investigation being reported | check |
| Objectives | 3 | State specific objectives, including any pre specified hypotheses | check |
| Methods | | |  |
| Study design | 4 | Present key elements of study design early in the paper | check |
| Setting | 5 | Describe the setting, locations, and relevant dates, including periods of recruitment, treatment, follow-up, and data collection | check |
| Participants | 6 | (*a*) *Cohort study*—Give the eligibility criteria, and the sources and methods of selection of participants. Describe methods of follow-up  *Case-control study*—Give the eligibility criteria, and the sources and methods of case ascertainment and control selection. Give the rationale for the choice of cases and controls  *Cross-sectional study*—Give the eligibility criteria, and the sources and methods of selection of participants | check |
|  |  | (*b*) *Cohort study*—For matched studies, give matching criteria and number of treated and untreated  *Case-control study*—For matched studies, give matching criteria and the number of controls per case |  |
| Variables | 7 | Clearly define all outcomes, exposures, predictors, potential confounders, and effect modifiers. Give diagnostic criteria, if applicable | check |
| Data sources/ measurement | 8* | For each variable of interest, give sources of data and details of methods of assessment (measurement). Describe comparability of assessment methods if there is more than one group | check |
| Bias | 9 | Describe any efforts to address potential sources of bias | check |
| Study size | 10 | Explain how the study size was arrived at | check |
| Quantitative variables | 11 | Explain how quantitative variables were handled in the analyses. If applicable, describe which groupings were chosen and why | check |
| Statistical methods | 12 | (*a*) Describe all statistical methods, including those used to control for confounding | check |
|  |  | (*b*) Describe any methods used to examine subgroups and interactions |  |
|  |  | (*c*) Explain how missing data were addressed | check |
|  |  | (*d*) If applicable, explain how loss to follow-up was addressed | check |
|  |  | (*e*) Describe any sensitivity analyses |  |
| **Results** |  |  |  |
| Participants | 13* | (a) Report numbers of individuals at each stage of study—eg, numbers potentially eligible, examined for eligibility, confirmed eligible, included in the study, completing follow-up, and analyzed | check |
|  |  | (b) Give reasons for nonparticipation at each stage | check |
| Descriptive data | 14* | (a) Give characteristics of study participants (eg, demographic, clinical, social) and information on other treatments and potential confounders | check |
|  |  | (b) Indicate number of participants with missing data for each variable of interest | check |
|  |  | (c) *Cohort study*—Summarize follow-up time (eg, average and total amount) | check |
| Outcome data | 15* | Report numbers of outcome events or summary measures over time | N/A |
| Main results | 16 | (*a*) Give unadjusted estimates and, if applicable, confounder-adjusted estimates and their precision (eg, 95% confidence interval). Make clear which confounders were adjusted for and why they were included |  |
|  |  | (*b*) Report category boundaries when continuous variables were categorized | N/A |
|  |  | (*c*) If relevant, consider translating estimates of relative risk into absolute risk for a meaningful time period | N/A |
| Other analyses | 17 | Report other analyses done—eg analyses of subgroups and interactions, and sensitivity analyses | N/A |
| Discussion |  |  |  |
| Key results | 18 | Summarise key results with reference to study objectives | check |
| Limitations | 19 | Discuss limitations of the study, taking into account sources of potential bias or imprecision. Discuss both direction and magnitude of any potential bias | check |
| Interpretation | 20 | Give a cautious overall interpretation of results considering objectives, limitations, multiplicity of analyses, results from similar studies, and other relevant evidence | check |
| Generalisability | 21 | Discuss the generalisability (external validity) of the study results | check |
| Other information |  |  |  |
| Funding | 22 | Give the source of funding and the role of the funders for the present study and, if applicable, for the original study on which the present article is based | check |

*Give information separately for cases and controls.

**Note:** An Explanation and Elaboration article discusses each checklist item and gives methodological background and published examples of transparent reporting. Information on the STROBE Initiative is available at <http://www.strobe-statement.org>.
